# Molecular Networking: A Useful Tool for the Identification of New Psychoactive Substances in Seizures by LC–HRMS

**DOI:** 10.3389/fchem.2020.572952

**Published:** 2020-11-25

**Authors:** Flaminia Vincenti, Camilla Montesano, Francesca Di Ottavio, Adolfo Gregori, Dario Compagnone, Manuel Sergi, Pieter Dorrestein

**Affiliations:** ^1^Department of Chemistry, Sapienza University of Rome, Rome, Italy; ^2^Department of Public Health and Infectious Disease, Sapienza University of Rome, Rome, Italy; ^3^Faculty of Bioscience and Technology for Food, Agriculture and Environment, University of Teramo, Teramo, Italy; ^4^Department of Scientific Investigation (RIS), Carabinieri, Rome, Italy; ^5^Collaborative Mass Spectrometry Innovation Center, Skaggs School of Pharmacy and Pharmaceutical Sciences, University of California, San Diego, San Diego, CA, United States

**Keywords:** new psychoactive substances, LC-HRMS, molecular networking, untarget analysis, seizure analysis

## Abstract

New Psychoactive Substances (NPS) are a global concern since they are spreading at an unprecedented rate. Despite their commerce still being limited compared to traditional illicit drugs, the identification of NPS in seizures may represent a challenge because of the variety of possible structures. In this study we report the successful application of molecular networking (MN) to identify unexpected fentanyl analogs in two seizures. The samples were extracted with 1 mL of methanol and analyzed with an untargeted data-dependent acquisition approach by LC–HRMS. The obtained data were examined using the MN workflow within the Global Natural Product Search (GNPS). A job was submitted to GNPS by including both seizures and standard mixtures containing synthetic cannabinoids and fentanyls raw files; spectra obtained from standards were used to establish representative networks for both molecular classes. All synthetic cannabinoids in the mixture were linked together resulting in a molecular network despite their different fragmentation spectra. Looking at fentanyls, all the molecules with the typical 188.143 and 105.070 fragments were combined in a representative network. By exploiting the standard networks two unexpected fentanyls were found in the analyzed seizures and were putatively annotated as para-fluorofuranylfentanyl and (iso)butyrylfentanyl. The identity of these two fentanyl analogs was confirmed by NMR analysis. Other m/z ratios in the seizures were compatible with fentanyl derivatives; however, they appeared to be minor constituents, probably impurities or synthetic byproducts. The latter might be of interest for investigations of common fingerprints among different seizures.

## Introduction

The most common approach used in forensic laboratories to identify and quantify illicit drugs in seized samples is targeted mass spectrometry (MS), usually coupled with Gas (GC) or Liquid Chromatography (LC). Targeted MS approaches are able to detect hundreds of illegal drugs at a trace level in a single analysis, in complex matrices such as biological samples or plant extracts (Archer et al., 2011; Smith et al., 2015). Identification and quantitation are carried out comparing retention times, fragmentation spectra and, for quantitative purposes, peak areas with analytical standards. Standards are generally very expensive and, sometimes, particularly for newly synthesized drugs and unknown metabolites, not commercially available (Laks et al., 2004).

These limitations of targeted analysis are exploited by drug producers to circumvent controls. This issue resulted in the New Psychoactive Substances (NPS) phenomenon. Several new drugs synthetized from known molecules by means of simple structural modifications such as alkylation, dealkylation, oxidation, reduction etc. were introduced in the market, becoming undetectable by traditional targeted screening. These new drugs are proliferating at an unprecedented rate, posing a significant risk to public health since they have unpredictable toxicological effects (Tai and Fantegrossi, 2014; Rivera et al., 2017; Weinstein et al., 2017).

The use of untargeted approaches is essential to solve this problem by exploiting suitable analytical tools; high resolution mass spectrometry (HRMS) is, at the moment, the most appropriate approach. From a theoretical point of view, having the accurate mass of a molecule and the isotopic pattern, the chemical formula of a compound can be confirmed or even ascertained, with a low rate of false positives. The study of the fragments may be an additional tool for the identification of unknown substances. Thus, HRMS, coupled with LC or GC for complex matrices, has been used to identify unexpected substances such as NPS in seized materials and metabolites in biofluids (Wille et al., 2017; Dei Cas et al., 2019). In seizures, HRMS can also represent an important tool to detect impurities and synthetic by-products to obtain a fingerprint of the samples. These signatures may be used to assess correlations among samples including their origin (Münster-Müller et al., 2019). HRMS has, then, become essential in forensic toxicology for the detection of unknown substances; however, the complexity of the data generated from the analysis of a sample is high and annotation is still a bottleneck, often limiting the use of the collected data. A number of proprietary or open software and platforms exist for the analysis of complex raw data from untargeted LC–HRMS analysis; they only allow the annotation of known compounds, through library searches (Hohrenk et al., 2020).

Molecular Networking (MN) is a computational strategy that may help visualization and interpretation of the complex data arising from MS analysis. MN is able to identify potential similarities among all MS/MS spectra within the dataset and to propagate annotation to unknown but related molecules (Wang et al., 2016). This approach exploits the assumption that structurally related molecules produce similar fragmentation patterns, and therefore they should be related within a network (Quinn et al., 2017). In MN, MS/MS data are represented in a graphical form, where each node represents an ion with an associated fragmentation spectrum; the links among the nodes indicate similarities of the spectra. By propagation of the structural information within the network, unknown but structurally related molecules can be highlighted and successful dereplication can be obtained (Yang et al., 2013); this may be particularly useful for metabolite and NPS identification.

MN has been implemented in different fields, particularly metabolomics and drug discovery (Quinn et al., 2017); MN in forensic toxicology was previously used by Allard et al. (2019) for the retrospective analysis of routine cases involving biological sample analysis. Yu et al. (2019) also used MN analysis for the detection of designer drugs such as NBOMe derivatives and they showed that unknown compounds could be recognized as NBOMe-related substances by MN.

In the present work the Global Natural Products Social platform (GNPS) was exploited to analyze HRMS/MS data obtained from the analysis of seizures collected by the Italian Department of Scientific Investigation of Carabinieri (RIS). The potential of MN to highlight and support the identification of unknown NPS belonging to chemical classes such as fentanyls and synthetic cannabinoids has been demonstrated.

## Materials and Methods

### Chemicals and Working Solutions

16 fentanyl derivatives and 16 synthetic cannabinoids were purchased from Cayman Chemical (Ann Arbor, Michigan, USA) and were used as reference compounds. The list is provided in Tables 1, 2. Methanol, ultrapure water, and acetonitrile were of HPLC grade and were purchased from Fisher Scientific Italia (Rodano, MI, Italy), while formic acid was from Sigma Aldrich (Milan, Italy). Two separate working solutions with a final concentration of 100 ng/mL in methanol were prepared from the stock solutions of the drugs and then stored at -20°C.

**Table 1:** List of synthetic cannabinoids included in the standard mixture.

**Analyte**	**Molecular formula**	**Exact Mass (m/z)**	**Fragment 1 (m/z)**	**Fragment 2 (m/z)**
UR-144	C_21_H_29_NO	312,2321	125,0960	214,1219
JWH-073	C_23_H_21_NO	328,1695	155,0488	200,1065
UR-144 N(4-hydroxypentyl)	C_21_H_29_NO_2_	328,2271	125,0959	230,1165
XLR-11	C_21_H_28_FNO	330,2227	125,0959	232,1128
JWH-018	C_24_H_23_NO	342,1852	155,0488	214,1221
AB-005	C_23_H_32_N_2_O	353,2587	112,1121	98,0966
JWH-122	C_25_H_25_NO	356,2008	169,0644	214,1221
N5-OH-JWH018	C_24_H_23_NO_2_	358,1801	155,0488	230,1178
N-COOH-JWH018	C_24_H_21_NO_3_	372,1594	155,0488	244,0959
JWH-081	C_25_H_25_NO_2_	372,1958	185,0593	214,1221
MAM2201	C_25_H_24_FNO	374,1914	169,0644	232,1126
AM-1220	C_26_H_26_N_2_O	383,2117	98,0966	112,1120
JWH-200	C_25_H_24_N_2_O_2_	385,1910	155,0488	114,0913
N-COOH-MAM2201	C_25_H_23_NO_3_	386,1750	169,06440	244,09620
N5-OH-JWH-081	C_25_H_25_NO_3_	388,1907	185,05930	230,11690
WIN-55	C_27_H_26_N_2_O_3_	427,2016	155,04880	100,07580

**Table 2:** Fentanyl derivatives included in the standard mixtures.

**Fentanyl derivative standard**	**m/z**	**Molecular formula**
**4-Anpp**	**281,2012**	**C**_**19**_**H**_**24**_**N**_**2**_
**Despropionyl Para-Fluorofentanyl**	**299,1918**	**C**_**19**_**H**_**23**_**FN**_**2**_
**Acetyl Fentanyl**	**323,2118**	**C**_**21**_**H**_**26**_**N**_**2**_**O**
**Acrylfentanyl**	**335,2118**	**C**_**22**_**H**_**26**_**N**_**2**_**O**
**Fentanyl**	**337,2274**	**C**_**22**_**H**_**28**_**N**_**2**_**O**
**α-Methylfentanyl**	**351,2431**	**C**_**23**_**H**_**30**_**N**_**2**_**O**
**Ortho-Fluorofentanyl**	**355,2180**	**C**_**22**_**H**_**27**_**FN**_**2**_**O**
Cis-3-methylthiofentanyl	357,1995	C_21_H_28_N_2_OS
**Ocfentanyl**	**371,2129**	**C**_**22**_**H**_**27**_**FN**_**2**_**O**_**2**_
**Furanylfentanyl**	**375,2067**	**C**_**24**_**H**_**26**_**N**_**2**_**O**_**2**_
Remifentanyl	377,2071	C_20_H_28_N_2_O_5_
**Butyryl-fentanyl-carboxy Metabolite**	**381,2173**	**C**_**23**_**H**_**28**_**N**_**2**_**O**_**3**_
Sufentanyl	387,2101	C_22_H_30_N_2_O_2_S
Alfentanyl	417,2609	C_21_H_32_N_6_O_3_

*Analytes in bold correspond to nodes in the fentanyl network*.

### Seizure Samples

Two seized samples (A and B) collected during investigative operations by RIS between September 2017 and December 2018 were analyzed by LC–HRMS. Since these samples are connected to criminal activity, it is not possible to provide further information such as location and exact data collection.

Seized samples were stored at room temperature until extraction, then 1 mg of seizure was extracted with 1 mL of methanol, vortexed for 1 min, sonicated at 25°C for 10 min and finally filtered through a 0.22 μm nylon filter from Agilent (Santa Clara, CA, USA). The obtained extracts are diluted 1: 10000 and subsequently 5 μL are injected into the UHPLC system.

### UHPLC–HRMS Analysis

Both the standard solutions and the seizures extracts were analyzed by UHPLC–HRMS. Ten μL of sample were injected into a UHPLC Dionex^TM^ UltiMate^TM^ 3000 Rapid Separation Liquid Chromatography (RSLC) system (Thermo Fisher Scientific, San Jose, CA, USA). Compounds separation was performed with a C18 Accucore™ column (30 x 2.1 mm) from Thermo Fisher packed with 2.6 μm spherical solid core ultrapure particles. Mobile phases were 10 mM ammonium formate + 0.1% formic acid in water (mobile phase A) and 0.1% formic acid in acetonitrile (mobile phase B) using a flow rate of 0.5 mL min^−1^. A linear gradient was applied in order to elute the compounds; from 0 to 1 min B was maintained at 5%, from 1 to 9 min B was increased to 100%, maintained at 100% for 2 min and afterwards 2.5 min re-equilibration at 5% B was performed. The C18 column was held at 40°C.

Analyses were performed with a Q Exactive Orbitrap MS from Thermo Fisher Scientific (Bremen, Germany) equipped with a Heated Electrospray Ionization (H-ESI) in positive mode. H-ESI conditions were set as follows: source temperature 340°C, capillary temperature 380°C, spray voltage 3.50 kV, S-lens RF level 60.0. Nitrogen was used for both sheath and auxiliary gas and was set at 60 and 20, respectively.

Untargeted analysis was conducted in Full MS/dd-MS2 acquisition mode, which combines a full scan with a set of data dependent MS2 scans. Full scan was carried out at a resolution of 35,000 (FWHM) in a scan range of 50–850 m/z. Automatic Gain Control (AGC) was 1e6 and Maximum Injection Time was 100 ms. MS/MS analyses were carried out with a resolution of 17,500 (FWHM), AGC and Maximum IT were set, respectively at 5e5 and 100 ms. Fragmentation was performed in HCD cell at three different values of normalized collision energy (NCE) 20, 30 and 40 with a dynamic exclusion of 30s, with nitrogen as collision gas.

### Data Processing

Raw data files obtained from the untargeted analysis of the selected samples and the standard mixtures were converted to .mzXML using MSConvert (http://proteowizard.sourceforge.net) in order to transform spectra from profile to centroid mode. The .mzXML files were uploaded on Global Natural Product Social Molecular Networking (GNPS) through WinSCP (version 5.17.3) and analyzed with the GNPS platform (http://gnps.ucsd.edu). For the MS-Cluster and spectral library search, parent ion mass and MS/MS fragment ion tolerance were set at 0.02 Da in order to create consensus spectra. Links between nodes were created when the cosine score was <0.7 and a minimum number of 6 common fragment ions were shared by at least one MS/MS spectrum. An exhaustive guide for MN building by means of GNPS was recently provided (Aron et al., 2020). Two separate jobs were carried out for synthetic cannabinoids and fentanyls, respectively, molecular networks are available at:

https://gnps.ucsd.edu/ProteoSAFe/status.jsp?task=3b4d5e5b455140ceb842d2aa13e51c1c

https://gnps.ucsd.edu/ProteoSAFe/status.jsp?task=193e9ab161554741a119940e2c52a2b0.

For each job, samples were separated into different groups, spectrum files of standard solutions were loaded as group 1 (G1), seizure A was labeled as G2 while seizure B as G3. For each group triplicate files were loaded. Library annotations were obtained from the comparison between the MS/MS spectra with several spectral libraries, including GNPS, NIST17, HMDB, and Massbank; at least 6 fragment ions should match the MS/MS spectra contained in those libraries with a cosine score of 0.7. The selected parameters allow a false discovery rate (FDR) of 1%, data obtained using Passatutto (Scheubert et al., 2017).

Finally, the resulting spectral network was uploaded in Cytoscape 3.8 to obtain better visualization, the nodes were labeled with ID and precursor masses; the edges with the mass differences between the connected nodes, and the edges thickness is proportional to cosine score. Nodes were colored in different colors according to the group where the precursor was detected.

All the software programs used in these steps are open source and can be accessed freely online.

### NMR Analysis

NMR experiments were recorded at 298 K on a AVANCE III spectrometer (Bruker BioSpin, GmbH, Germany), equipped with a multinuclear z-gradient inverse probe-head operating at the proton frequency of 400.13 MHz.

Assignments were made via ^1^H NMR as well as bidimensional ^1^H/^1^H correlation spectroscopy (^1^H/^1^H–COZY) and ^1^H/^13^C–heteronuclear single-quantum correlation spectroscopy (^1^H/^13^C–HSQC). Spectra were analyzed with ACD NMR manager software ver. 12 (ACD/Labs, Toronto, Canada).

## Results and Discussion

The aim of this study was to exploit MN to process the data obtained from the analysis of seizures using HRMS/MS in untargeted acquisition mode. The GNPS platform is helpful for investigation purposes related to drug seizures because of the ability to annotate illicit drugs even in the absence of standards. This is achieved by exploiting the available libraries and propagating, at the same time, the annotation to structurally related substances by MN, which is very promising for NPS identification.

Initially LC-HRMS chromatographic parameters were adjusted in order to obtain an optimal separation and peak shape for the analytes included in the standard mixtures. Being the method aimed to the detection of molecules not included in the target list, further adjustments to the method were necessary in order to allow retention of compounds in a wider polarity range with respect to the standards. The Full MS/dd-MS2 acquisition mode, which combines a full scan with a set of data dependent MS2 scans, provides a unique tool for untargeted analysis. Then a fine tuning was made on the HRMS parameters in order to obtain the best sensitivity together with a suitable resolution and accuracy: full scan and MS/MS resolution, AGC, injection time and fragmentation conditions were adjusted.

The LC–MS/MS raw files arising from the analysis of the standard mixtures and the seizure samples were separated in three groups as described in §2.4 and submitted to GNPS. The obtained networks allowed the visual exploration of compound families, within the different samples. Each node had a distinctive color based on the group to which they belonged. When a query MS/MS spectrum matched a GNPS library entry, the node was highlighted.

### Synthetic Cannabinoids Network

GNPS spectral libraries were able to identify 5 of the 16 drugs included in the standard mixture; no relevant matches were identified in the seizures (G2 and G3). Annotation was carried out based on the exact mass and the match between the fragmentation spectrum and the GNPS spectral databases.

**Figure 1:**
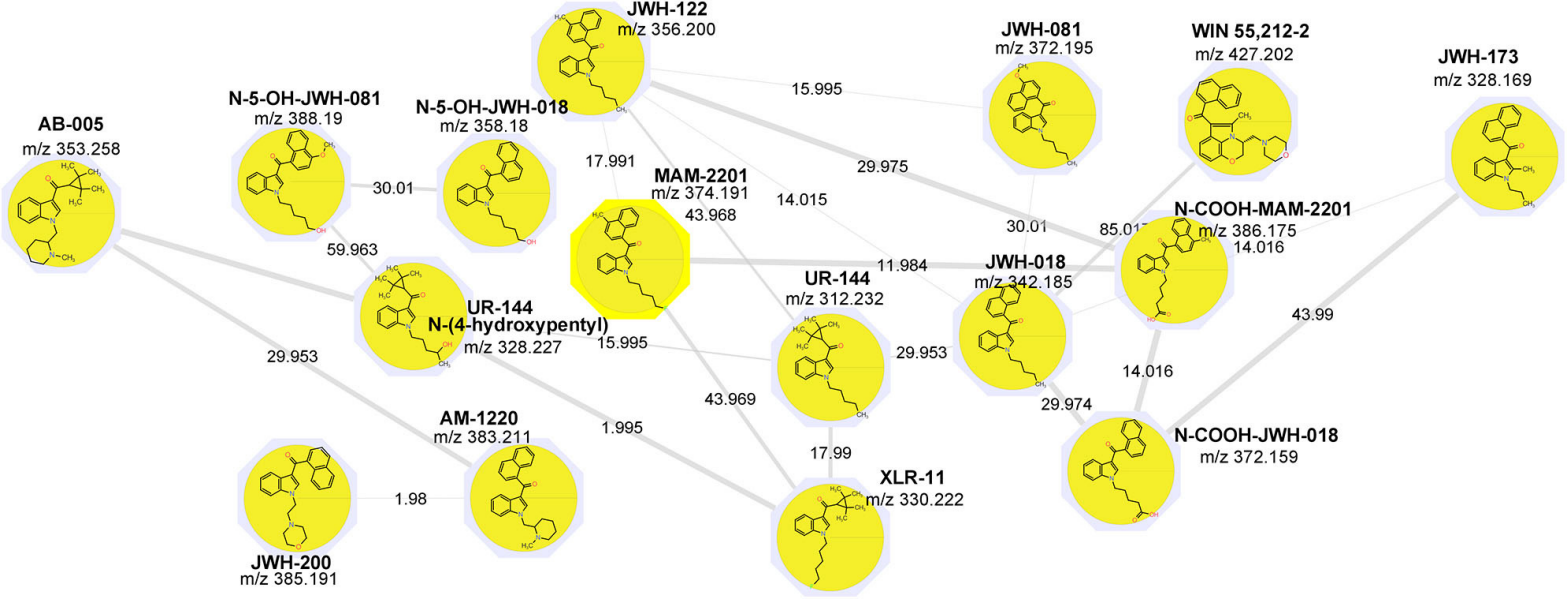
Synthetic cannabinoids network.

Attention was then paid to the spectral families. A large network of 31 nodes was identified as the synthetic cannabinoids network; it was remarkable that all 16 cannabinoids included in the sample were recognized as one integrated network with cosine score > 0.7 (Figure 1). The network also contained additional nodes that did not correspond to the added standards. It was observed that most of these “unknowns” had a lower intensity; then after applying a filter on precursor intensity (1E7), only 9 of them were kept. Examining the data, it was observed that all the nodes were correlated to precursors with a mean R_t_ within 1% of the standards suggesting that they were adducts and fragments of the standards. Notably, the precursor charge of most unknown was 0 and their precursor mass corresponded to [M-1]. All these nodes were excluded from the network, the final network is shown in Figure 1.

Interestingly, analytes not sharing common fragments were connected within the network, nicely showing the potential of MN to reveal new synthetic cannabinoids that cannot be identified using conventional approaches such as precursor ion or neutral loss scanning. In fact, MN not only groups fragmentation spectra (MS2) with ions at identical m/z, but also MS2 that are offset by the same m/z difference as the precursor ion. MS/MS structural similarity is expressed by the cosine scores for the vectors generated from an m/z value and the respective intensity of the product ions. In the present study the aim was to construct a “training network” with standards and to exploit it to possibly annotate unknown seizure samples. In fact, considering that related molecules are connected into the same subnetwork, even unknown but structurally related compounds will be included in this cluster, offering an interesting perspective to annotate new synthetic cannabinoids.

Raw files were then separated in different groups; group 1 included the standards while group 2 and 3 the seizures. The two seizures were previously analyzed by a target LC-HRMS method including nearly 50 NPS but no positivities were found. No synthetic cannabinoids were found in the seizures using the MN-based screening method.

### Fentanyl Network

**Figure 2:**
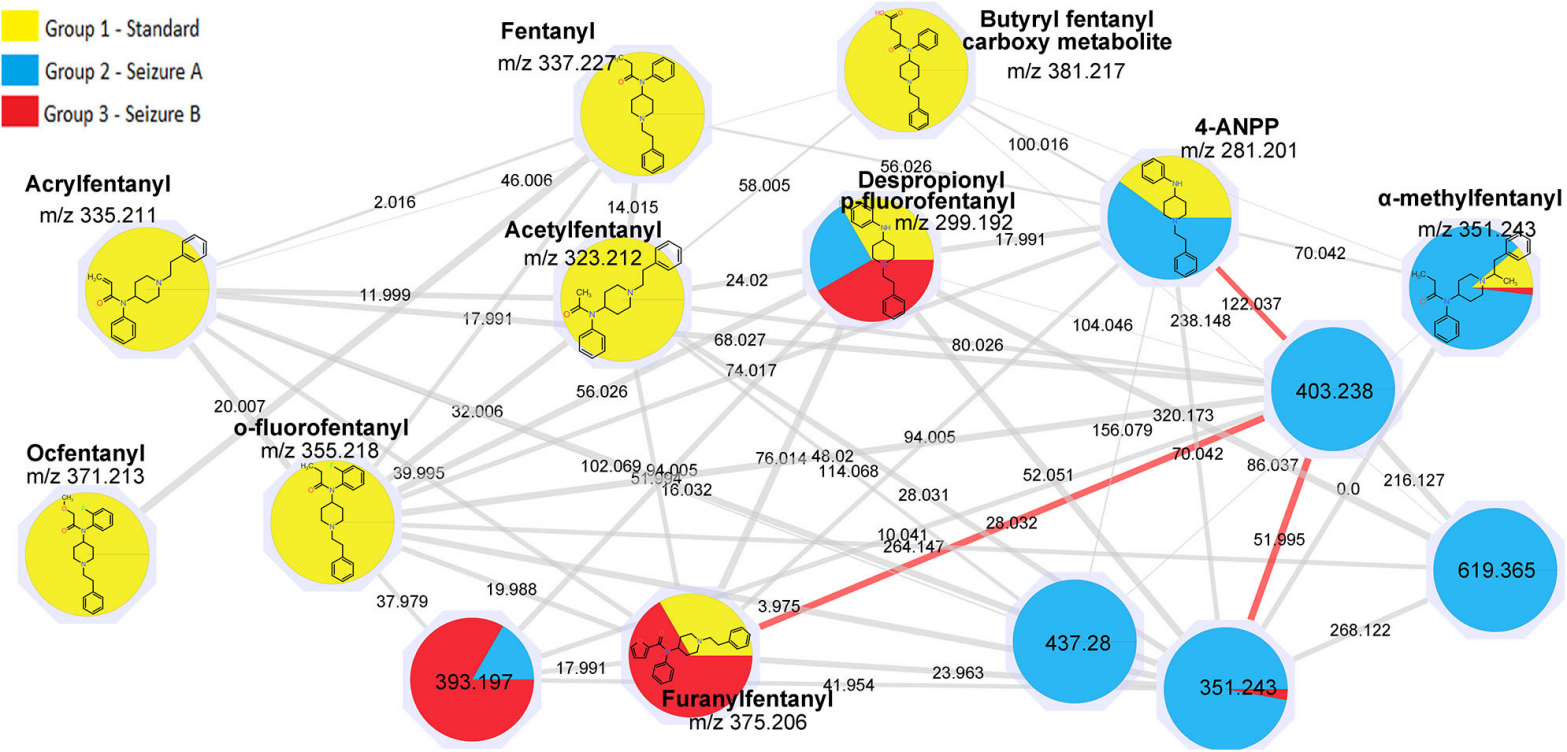
Fentanyl network.

For fentanyls, 4 out of 16 compounds of the mixture were correctly identified by library matching, namely fentanyl (identified as “Innovar” which is a trade name of a drug containing fentanyl) remifentanyl, sufentanyl, and alfentanyl while no hits were found for the remaining drugs. Among the drugs identified by the library matching, only fentanyl (m/z 337.227) was included in a network (Figure 2). The original network contained 25 nodes, however, as reported above for synthetic cannabinoids, nodes with lower intensity and nodes that were likely to belong to adducts and in-source fragments were deleted; the cleaned network included 16 nodes.

**Figure 3:**
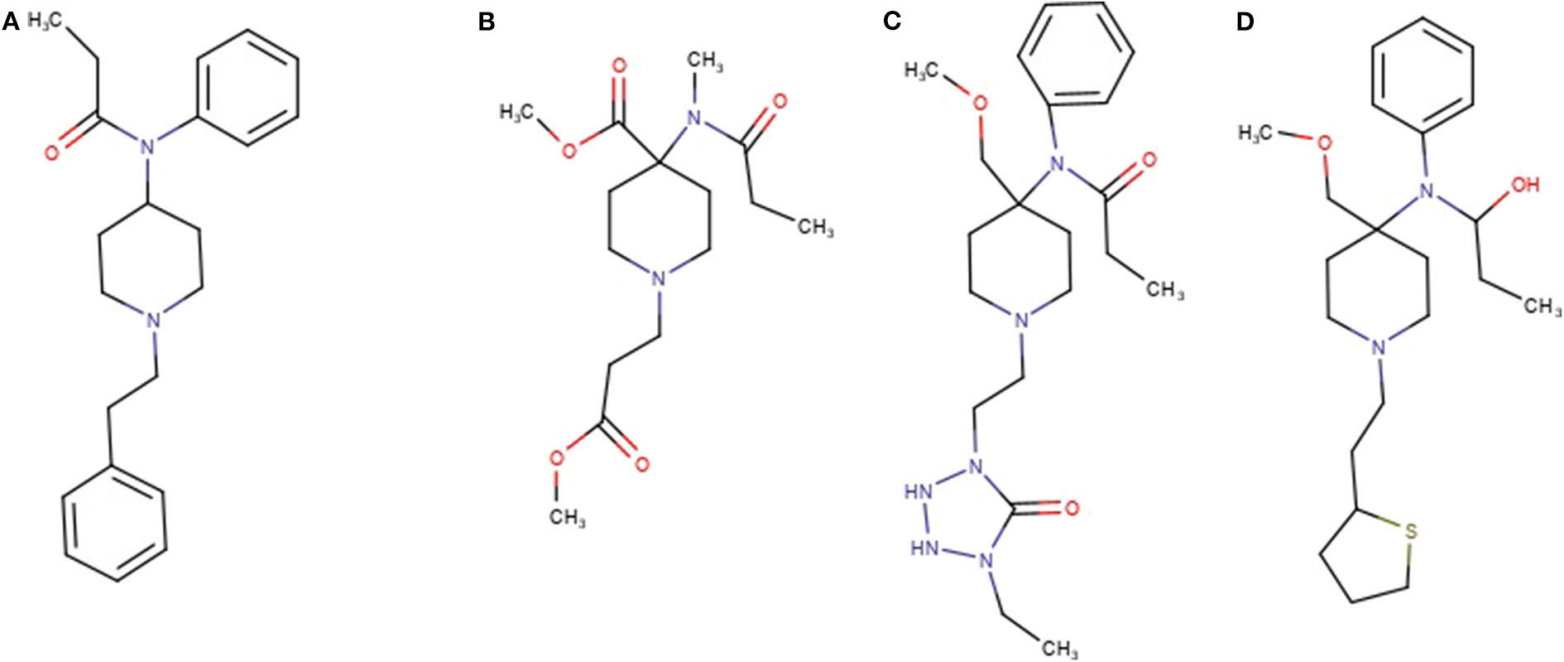
Structures of Fentanyl **(A)**, remifentanyl **(B)**, alfentanyl **(C)**, and sufentanyl **(D)**.

Ten of the standards included in the mixture were found in this network as reported in Table 2. These drugs correspond to the yellow colored nodes, while nodes from group 2 are colored in blue and group 3 in red. From a visual analysis it can be noticed that nodes belonging to this network are from the three groups, suggesting that seizures A (group 2) and B (group 3) contained fentanyl derivatives. In the case of fentanyls, differently from synthetic cannabinoids not all the standards of the mixture were included in the network. This can be explained observing the molecular structures of the identified fentanyls (Figure 3) which lead to completely different fragmentation spectra. Fentanyls not included in the network were those not having the typical phenylethyl piperidine moiety which gives rise to MS2 spectra with the characteristic m/z 188.143 and 105.070 fragments.

This results in showed a limitation of MN for the identification of fentanyl derivatives with a different base structure. Future algorithms may be able to find additional connections, even when the overall fragmentation behavior is altered, as long as some overlap in fragmentation is present.

However, good data were obtained for the seizures; some unknown hits were connected to the standard fentanyl network suggesting that they were structurally related to the standards, with a cosine score >0.9. A number of nodes were exclusively found in the seizures while some nodes were common to seizures and standard mixture as listed in Table 3.

**Table 3:** Nodes exclusively found in the seizures and nodes common to seizures and standard mixture.

**Node Precursor m/z**	**Node Rt (s)**	**Group**	**ID**
403.238	230.90	Seizure A	*dimethylfuranyl-fentanyl*
619.365	317.06	Seizure A	unknown
437.280	242.78	Seizure A	unknown
351.243	409.30	Seizure A and standard mix	α-methylfentanyl
281.201	180.26	Seizure A and standard mix	4-ANPP
351.243	212.88	Seizure A and B	*(iso)butyrylfentanyl\*
393.197	188.91	Seizure B and A	*Para-fluorofuranylfentanyl*
299.192	143.84	Seizure A and B + standard mix	despropionyl para-fluorofentanyl
375.206	142.75	Seizure A and B + standard mix	furanylfentanyl

In addition to the identification of unexpected drugs, an important feature of MN analysis is that the structures of the unknowns may be hypothesized, based on the precursor mass difference between nodes. The node with mass 299.192 corresponded to despropionyl para-fluorofentanyl, which is known to be a metabolite of 4-fluoroisobutyryl fentanyl, but also a precursor in the synthesis of para-fluorofentanyl; the presence of this compound in both seizures suggested that they contained a para-fluorofentanyl derivative. From the analysis of the network, it can be noticed that despropionyl para-fluorofentanyl is connected to the node with m/z 393.197 (cos 0.95) which was found in both seizures and was among the most intense peaks in seizure B. The delta mass between these two nodes was 94.005, which correspond to a C_5_H_2_O_2_ moiety; the node with m/z 393.197 was also connected to furanylfentanyl node with a cosine score of 0.93 and, in this case the delta mass between these two nodes was 17.991 which possibly arose from the addition of a fluorine and the loss of a proton. This observation suggested that peak with m/z 393.197 is a fluoro-furanylfentanyl; the presence of despropionyl para-fluorofentanyl in the seizures indicate that it could be para-fluorofuranylfentanyl, arising from the addition of a furanyl moiety (C_5_H_3_O_2_) to the precursor despropionyl para-fluorofentanyl.

**Figure 4:**
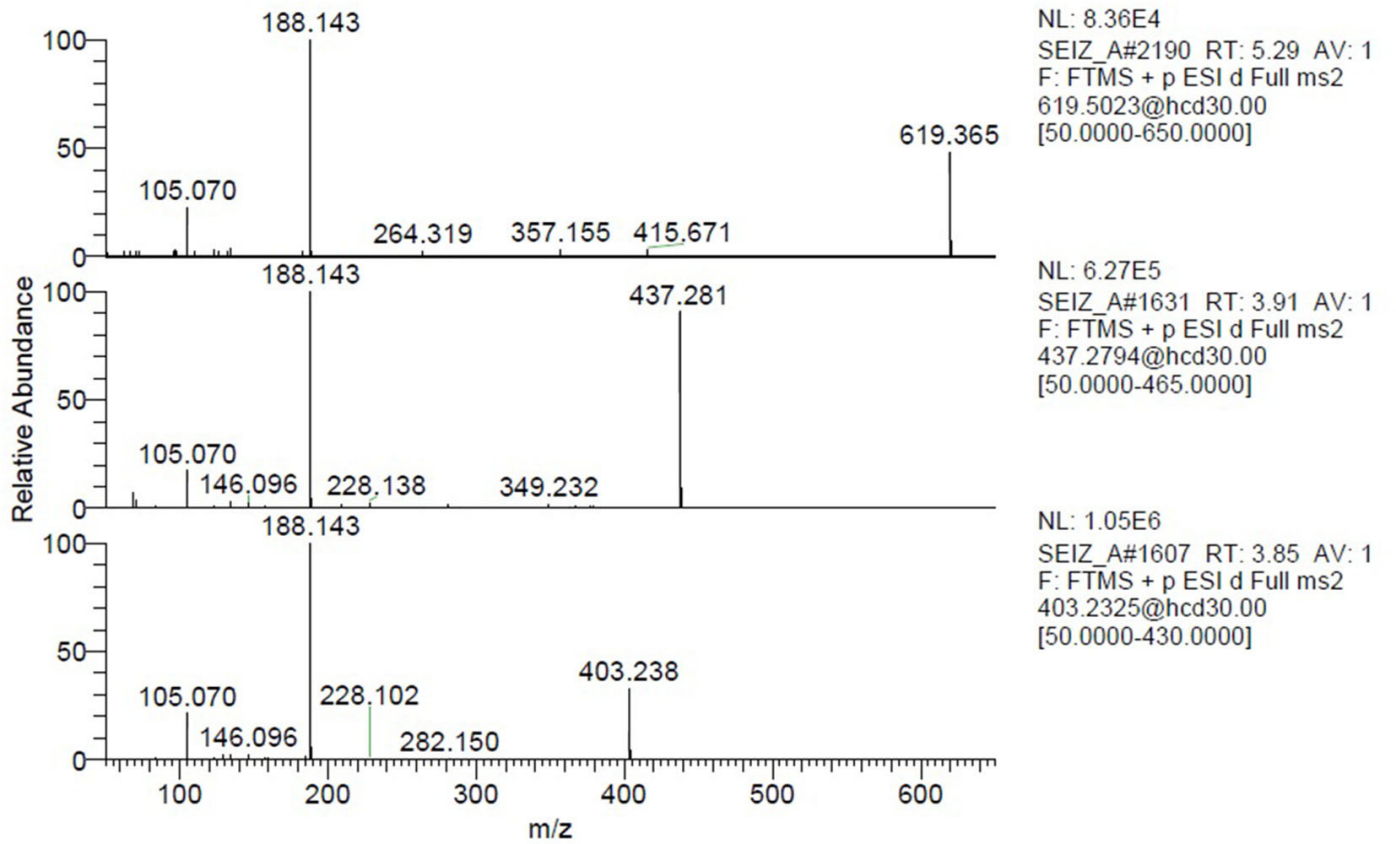
MS/MS spectra of the two nodes with precursor m/z = 351.243.

Another interesting observation was that two different nodes had m/z 351.243. One of these corresponded to α-methylfentanyl which was among the selected standards. In fact, it was found in group 1, the corresponding spectrum showed the characteristic fragment 202.159 (Figure 4) which arose from the loss of the methylated phenylethyl piperidine moiety. The spectrum of the node which shared the same mass of α-methylfentanyl is different and only the typical m/z 188 and 105 fragments are present, suggesting that the phenylethyl piperidine moiety was unmodified; this compound, which was found in both seizures and mainly in seizure A, can be putatively annotated as butyrylfentanyl or isobutyrylfentanyl.

**Figure 5:**
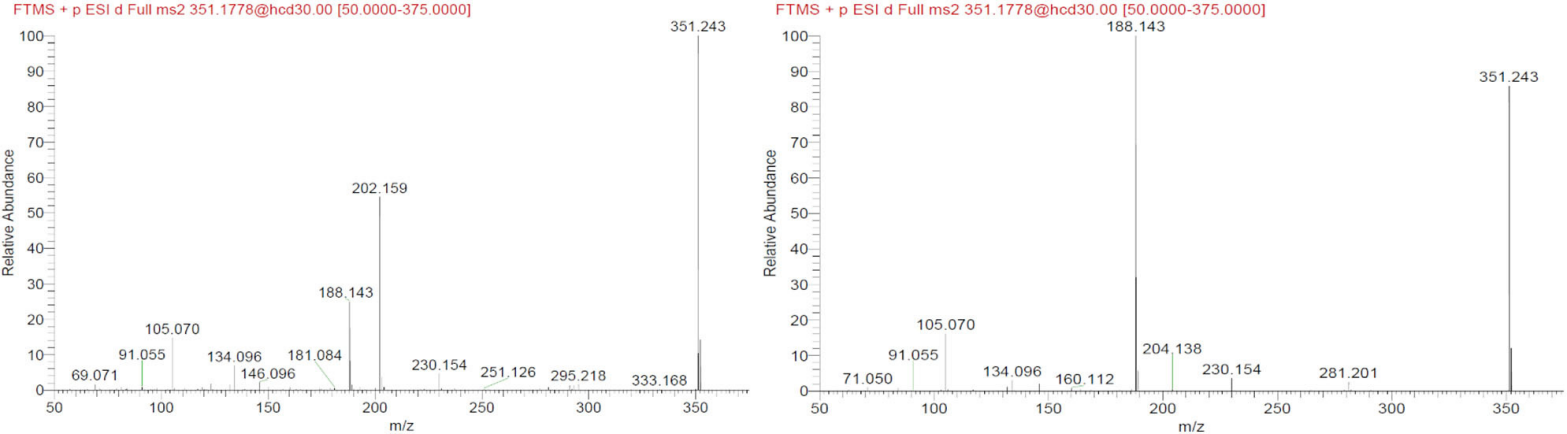
Spectra of the unknown nodes with precursor m/z = 619.365, 437.281, and 403.238.

Seizure A also contained 4-ANPP which is a well-known fentanyl precursor (Drug Enforcement Administration, 2010) and 3 unknown compounds, namely m/z 437.280, 619.365, and 403.238 whose spectra are shown in Figure 5. All three spectra are compatible with fentanyl derivatives on the basis of the presence of the characteristic m/z 105 and 188 fragments, but the precursor masses do not match with any known fentanyl. Compared with the peaks putatively annotated as butyrylfentanyl and 4-Fluoro-furanylfentanyl, their intensity is 2–3 order of magnitude lower. Interestingly the node with mass 403.238 is central in the fentanyl network, supporting the thesis that it may be a fentanyl derivative. The node is linked to 4-ANPP (Δmass 122.037, C_7_H_6_O_2_), furanylfentanyl (Δmass 28.032, C_2_H_4_) and butyrylfentanyl (Δmass 51.995, C_3_O) among others, with cosine score >0.9. By taking into account the delta masses between nodes (Figure 2) and the fragmentation spectrum a possible assignation could be dimethylfuranyl-fentanyl.

A hypothesis is that the three unknown compounds in seizure A are synthetic byproducts and even if it was not possible to assign a possible structure for all these compounds at this stage, the detection of possible impurities is a tool for investigations since they might be an important marker for its source and are likely to be specific for a particular synthesis site or wholesaler.

### NMR Analysis of Seizures

The fentanyls in the seized samples were finally identified by ^1^H and ^13^C NMR spectroscopy (including 2D experiments).

#### 4-Fluorofuranyl Fentanyl

Monodimensional and homonuclear ^1^H-^1^H TOCSY experiments allowed for the identification of the spin systems which include aromatic protons and three protons of the furan moiety at 7.36, 6.22, and 5.61 ppm, respectively. Moreover, it was possible to observe the presence of another seven non-magnetically equivalent CH groups in the aromatic region on the basis of heteronuclear ^1^H-^13^C HSQC. Due to the molecule symmetry, 3 of these resonances belong to the protons 2'-6', 3'-5', and 4' of the benzene ring in the phenylethylpiperidin moiety. Instead, the fluorophenyl moiety is bonded to a tetrahedric nitrogen atom, implying the loss of molecular symmetry in this group; given the similarity in the chemical shifts observed in the spectrum, the fluoride must be in position 4 to reach the total number of non-equivalent carbons.

#### Isobutyrylfentanyl

The presence of iBF was also confirmed by NMR, the spectra showed the typical signals of fentanyl analogs while the existence of the iso-butyl moiety was confirmed by the presence of a doublet at 0.91 ppm in the proton spectrum (data not shown). This multiplicity was not possible for a CH_3_ belonging to a linear chain, but only to an iso-butyl one.

## Conclusions

The data reported demonstrated the potential of GNPS in the forensic field particularly for NPS analysis. In fact, library matching with crowdsourced databases may allow the annotation of unexpected compounds, on the other hand MN allows to connect unknown compounds to “standard networks,” simplifying the annotation of new drugs. In the reported cases, putative assignment of modifications was possible on the basis of the precursor mass difference. Two previously unidentified fentanyls were found in the analyzed seizures. They were putatively identified as para-fluorofuranylfentanyl and (iso)butyrylfentanyl by connections to fentanyl standard network nodes. To confirm these annotations the samples were analyzed by NMR. It was also shown that structurally related compounds that do not share common fragments, such as some synthetic cannabinoids, formed an integrated network.

This study demonstrates that GNPS is a very useful tool in forensic investigations particularly for identification of new drugs and metabolites. In future, MN could represent an important tool in the forensic field, but to reach its full potential the public sharing of data is needed.

## Data Availability Statement

The datasets presented in this study can be found in online repositories. The names of the repository/repositories and accession number(s) can be found below: https://gnps.ucsd.edu/ProteoSAFe/status.jsp?task=3b4d5e5b455140ceb842d2aa13e51c1c; https://gnps.ucsd.edu/ProteoSAFe/status.jsp?task=193e9ab161554741a119940e2c52a2b0.

## Author Contributions

FV: investigation and data acquisition. CM: writing—original draft, data analysis, and conceptualization. FD: data analysis. AG: project administration and supervision. DC: supervision and writing-reviewing and editing. MS: conceptualization, methodology, and writing–original draft and editing. PD: conceptualization and editing. All authors contributed to the article and approved the submitted version.

## Conflict of Interest

The authors declare that the research was conducted in the absence of any commercial or financial relationships that could be construed as a potential conflict of interest.
